# Pathophysiology and Biomarkers in Rheumatoid Arthritis Patients: Immunological and Erythrocyte Sedimentation Rate Alterations

**DOI:** 10.12688/f1000research.174118.1

**Published:** 2025-12-26

**Authors:** Mohammed Y.I. Al-Hamadani, Hussain Abdalwahab Bdewi, Ashjan Mohammed Hussein, Anwar Khalil Ismael, Leqaa M Aziz, Mokhtar I Yousef

**Affiliations:** 1 Department of Biology, University of Fallujah, Al-Fallujah, Al Anbar Governorate, 00964, Iraq; 2 Department of Microbiology, College of Medicine, University of Fallujah, Al-Fallujah, Al Anbar Governorate, 00964, Iraq; 3 Department of Microbiology, University of Fallujah, Al-Fallujah, Al Anbar Governorate, 00964, Iraq; 4 Department of experimental therapy, Mustansiriyah University, Baghdad, Baghdad Governorate, 00964, Iraq; 5 Department of Pathological Analysis, University of Fallujah, Al-Fallujah, Al Anbar Governorate, 00964, Iraq; 6 Department of Environmental Studies, Institute of Graduate Studies and Research, Alexandria University, Alexandria, Alexandria Governorate, 832, Egypt

**Keywords:** Rheumatoid Arthritis, Anti-CCP Ab; Anti-MCV Ab; Rheumatoid Factor RF-titration; C-Reactive Protein C-RP; Immunoglobulin IgG and IgM; Erythrocyte Sedimentation Rate.

## Abstract

**Background:**

Rheumatoid arthritis (RA) is an autoimmune condition characterized by symmetrical and chronic pain and inflammation, initially affecting the small joints of the hands and feet, and later the larger joints. RA also attacks other organs of the body, particularly the skin, eyes, heart, kidneys, and lungs.

**Aim:**

The purpose of this research is to assess and characterize the hematological and immunological parameters that indicate the presence of inflammation and autoimmune conditions in persons with RA.

**Methods:**

90 male participants were recruited from several healthcare facilities, including Fallujah Teaching Hospital and Diagnostic Laboratories. The participants, RA patients (60) and healthy controls (30) were distributed into 2 groups for the comparative analysis of the hematological and immunological parameters.

**Results:**

Significant elevations (P < 0.0001) in erythrocyte sedimentation rate and immunoglobulins G and M were observed in RA patients across three age groups: young adults (25–40 years), middle-aged (41–65 years), and older adults (66–90 years). Anti-cyclic citrullinated peptide, anti-mutated citrullinated vimentin, rheumatoid factor, and C-reactive protein showed significant increases (P < 0.0001) across all age categories.

**Conclusion:**

The results reveal systemic inflammation and autoimmune dysregulation that are independent of patient age. Using immunological and hematological biomarkers in routine practice can improve diagnostic accuracy, guide personalized treatment, and help prevent joint damage while enhancing patients’ quality of life.

**List of abbreviations**
Anti-MCV
: Anti-mutated citrullinated vimentin
Anti-CCP
: Anti-cyclic citrullinated peptide
CRP: C-Reactive Protein
ESR: Erythrocyte Sedimentation Rate
IgG: Immunoglobulin
IgM: Immunoglobulin
RA: Rheumatoid Arthritis
RF: Rheumatoid Factor

## Introduction

Rheumatic arthritis (RA) is one of the common autoimmune, chronic debilitating systemic inflammatory diseases, mainly targeting synovial joints. Its systemic manifestations, affecting multiple organ arthritis, and extra and extra-articular RA also leads to large systemic burden on the patient and the population. RA also has the largest burden of loss to follow up and poor adherence to therapy of all the chronic systemic diseases ( Bullock et al., 2019). Women are affected three times more than men ( Venetsanopoulou et al., 2023). Apart from the synovial joints attacking tendons and ligament, RA leads to disabling bone and cartilage erosion. A large range of bone destruction and systemic diseases like skin, eye, heart, kidney, and lungs, are affected by the chronic escalating inflammatory nature of the disease. RA adversely and disproportionately affects the women, constituting the largest burden of chronic diseases globally.

The large and incompletely understood factors affecting the range of observed global prevalence of RA include poorly defined study population, poorly defined case finding for RA, differences in data source, sample size, study year range, and differing classification schemes for classifying RA ( Almutairi et al., 2021).

RA limits one’s potential in the workplace and affects mobility. Those living with RA have been found to have a reduced life expectancy when compared to the general population. The risk of mortality increases with the severity of RA due to debilitating damage and extensive radiography, particularly with poor functional status. Comorbidities, socioeconomic and educational status, and treatment accessibility predicated on socioeconomic factors influence RA mortality to a great extent and may be countered with the right strategies for disease management. Severe infections and high-dose corticosteroids, which are often used and switched on intermittently, greatly contribute to the risk of dying ( Almoallim et al., 2021).

Research on hematological indices as a means of gauging treatment response to both traditional and biological disease-modifying antirheumatic drugs (DMARDs) is limited ( Choe and Kim, 2022). The intro at high specificity in the mid-90s of the anti-cyclic citrullinated peptide (anti-CCP) antibody test provided a serological means to improved dx of RA, particularly in early disease ( Nielen et al., 2004).

In connection with this, early research showed an association between the presence of anti-CCP antibodies and the severity of early RA ( De Rycke et al., 2004). Early and timely treatment of RA, particularly with poor prognostic indicators, is of utmost importance to maximize outcomes ( Black et al., 2023). In the past, the first signs of RA involved the development of swollen joints. Nowadays, RA is considered a ‘disease continuum’ and is better understood. The RA disease continuum theory presents the idea that for certain individuals with a genetic or environmental predisposition for the disease, the disease starts with a subclinical phase which involves an autoimmune and subclinical inflammatory process followed by a chronic and clinically obvious phase ( Smolen et al., 2018).

Having RA-related autoantibodies tends to suggest a more aggressive disease course with rapidly destructive arthritis, high disease activity, and extra-articular manifestations ( Westerlind et al., 2023).

A 2021 study conducted by Molander and colleagues revealed a strong relationship between different indicators of RA disease activity and the likelihood of venous thromboembolism (VTE) in the following year, irrespective of RF and anti-citrullinated protein antibodies (ACPA) ( Molander et al., 2021). The literature has noted a short-term increase in VTE risk after the commencement or change of disease-modifying antirheumatic drugs (DMARDs). Since numerous patients change DMARDs due to poorly controlled disease, this circumstance adds to the evidence of the link between poorly controlled RA and the risk of VTE ( Ozen et al., 2021). The current study seeks to describe the pathogenesis of RA in infected patients. To describe the clinical and pathological findings of RA in infected patients. To assess the role of infection in the activity and course of RA. To find candidate biomarkers of RA in infected patients for disease activity or response to therapy. To find candidate therapeutic targets to enhance the control of RA in infected patients.

## Materials and methods

In this study, we collected 90 male cases from various healthcare settings, Fallujah Teaching Hospital and Diagnostic Laboratories (Only males were selected to avoid the effect of hormonal changes in females on immune and blood parameters, with the aim of obtaining more accurate and clear results, with future studies to include a comparison between the sexes.). The samples were divided into two main groups. The first main group was used healthy individuals as a control group (n=30 cases) and divided into three subgroups: Group 1, aged from 25-40 years (n=9 cases), Group 2, aged from 41-65 years (n=11), and Group 3, aged 66-90 years (n=10). The second main group of early diagnosed with RA without treatment (n=60 Patients) was divided into three subgroups: Group 1, age 25-40 years (n=17 cases), Group 2, age 41-65 years (n=21), and Group 3, age 66-90 years (n=22). All participants were diagnosed based on clinical examination by a Rheumatologist, and the selection criteria followed the ACR/EULAR 2010 Rheumatoid Arthritis Classification Criteria ( Aletaha et al., 2010). Participants with other chronic diseases, such as cardiovascular, hypertension, diabetes type I, II, or any other chronic autoimmune diseases, were excluded from this study. Blood samples (5 ml) were collected in a gel tube and left at room temperature for 15 min. Blood tubes were centrifuged at 860×g for 10 min. Serum was collected and stored at -20 °C until used. Parameters were measured by fully automated immunological tests by Alegria ^®^ ORGENTEC – Germany for (anti-CCP Ab, anti-MCV Ab, Rheumatoid Factor titration). The total immunoglobulin screening (IgG & IgM) and C-Reactive Protein tests were measured by Dimension ^®^ EXL 200 – Germany. ESR was measured by manual protocol. The experiment was conducted during the period from July 2023 to October 2023.

### Statistical analysis

Software Prism Graph Pad 8.0.1 was used for statistical analysis. A t-test was performed to find the significance between the average values of patients and healthy controls. All the compared values were shown to be significant. P value was considered P < 0.05.

## Results

**Figure 1.**
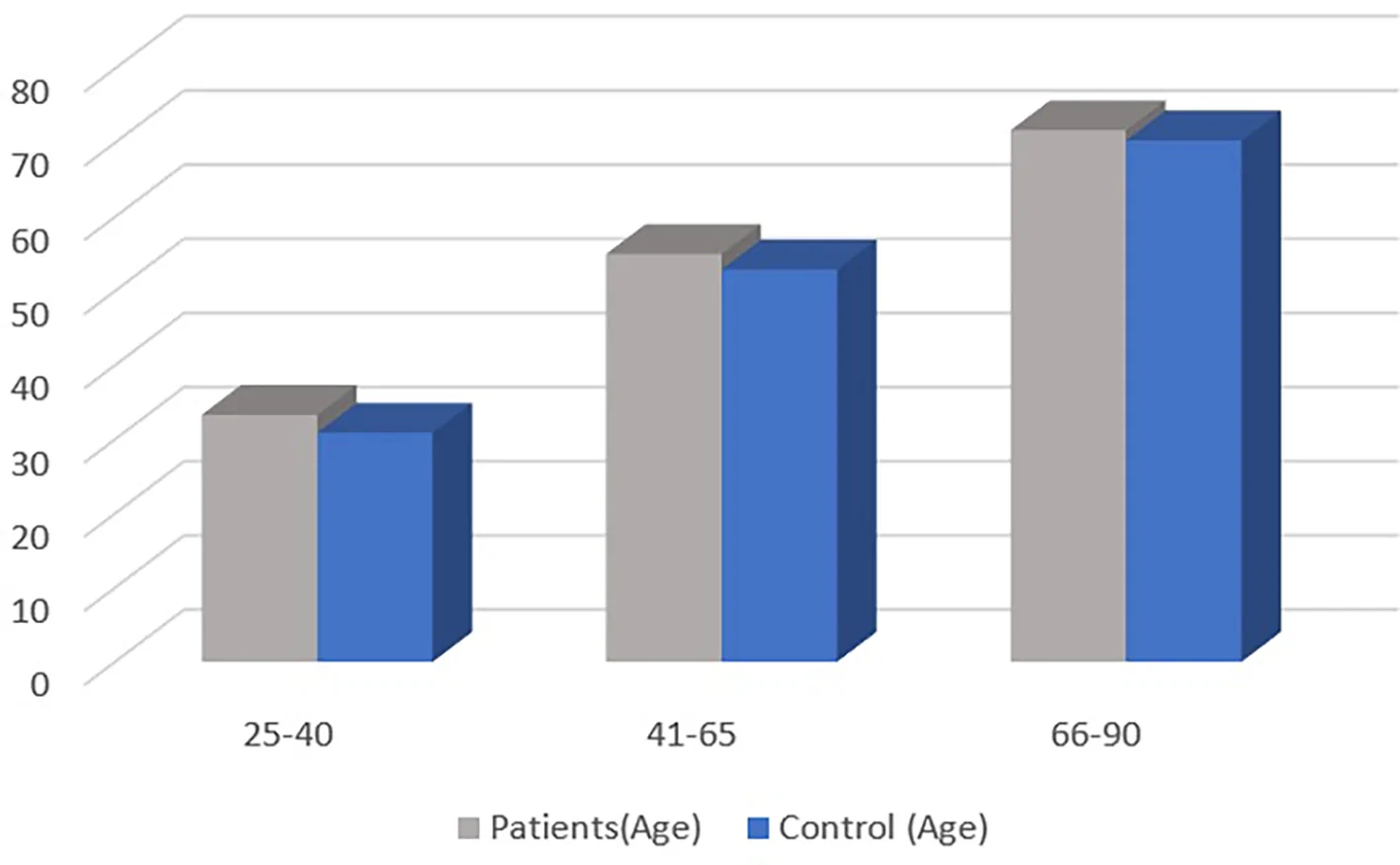
The ages of patients with RA and controls.

There were considerable age differences noted for Rheumatoid Arthritis (RA) patients compared to the control groups for the age brackets of 25-40, 41-65, and 66-90. The average ages of RA patients were significantly older compared to their control groups for all subgroups (P < 0.0001), and significant age-related differences (P < 0.0001) were noted for all subgroups for both RA and control groups. These differences are summarized in Table 1 and Figure 1.

**Table 1.** The ages/years of patients with Rheumatoid Arthritis (RA) and controls for different subgroups of age (Means ± SD).

Group Age (y)	No. of samples	Patients		No. of samples	Control		P value
		Mean	SD		Mean	SD	
25 – 40	17	33.35	4.91	9	30.89	4.91	<0.0001 *
41 – 65	21	55.00	5.83	11	52.91	8.04	<0.0001 *
66 – 90	22	71.68	5.81	10	70.30	4.46	<0.0001 *

* Indicate statistically significant differences at P < 0.0001.

**Figure 2.**
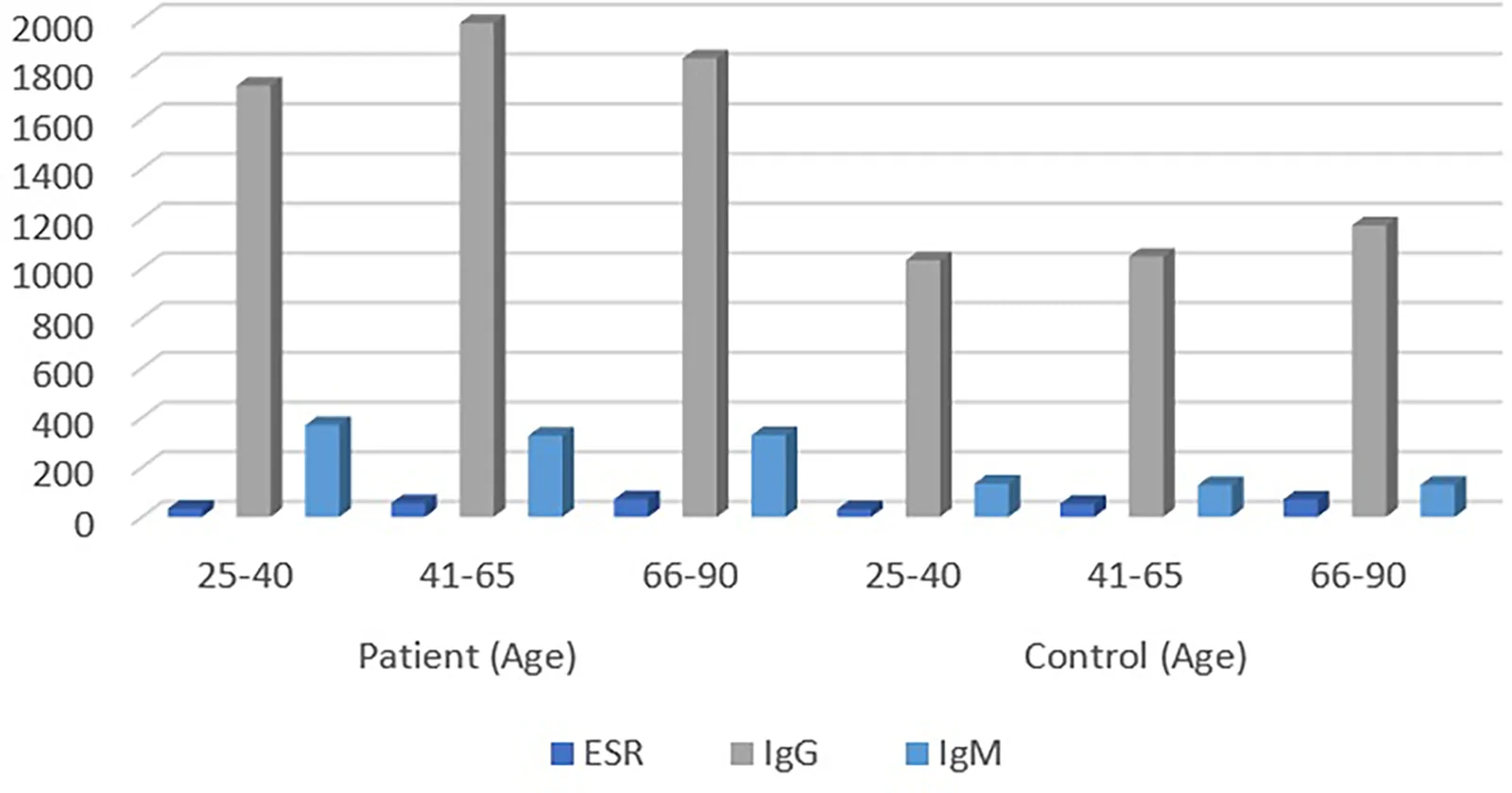
ESR, IgG, IgM of patients with Rheumatoid Arthritis (RA) and controls.

The results for the ESR, IgG and IgM are shown for the RA subgroups and their corresponding controls in Table 2 and Figure 2. The results showed a statistically significant (P < 0.0001) increase in ESR for all RA subgroups. However, for the remaining parameters, the RA and control groups in each subgroup did not demonstrate significant differences.

**Table 2.** Erythrocyte Sedimentation Rate (ESR), Immunoglobulin (IgG), and Immunoglobulin (IgM) of patients with Rheumatoid Arthritis (RA) and controls for different subgroups of age.

Parameter	Groups						P value
	Patient (Age/y)			Control (Age/y)			
	25-40	41-65	66-90	25-40	41-65	66-90	
	Means±SD	Means±SD	Mans±SD	Means±SD	Means±SD	Means±SD	
** ESR(mm/hr)**	33.4±4.9	55.0±5.83	71.68±5.81	30.9±4.9	52.9±8.04	70.3±4.46	<0.0001 *
**IgG(mg/dl)**	1735±496	1987±261	1845.0±435	1032±105	1047±227	1173±241	<0.0001 *
**IgM(mg/dl)**	369±70.7	327±63.1	328.6±62.53	134±37.9	128±49.8	130±47	<0.0001 *

ESR: Erythrocyte Sedimentation Rate, IgG: Immunoglobulin G, IgM: Immunoglobulin M.

* Indicate statistically significant differences at P < 0.0001.

**Figure 3.**
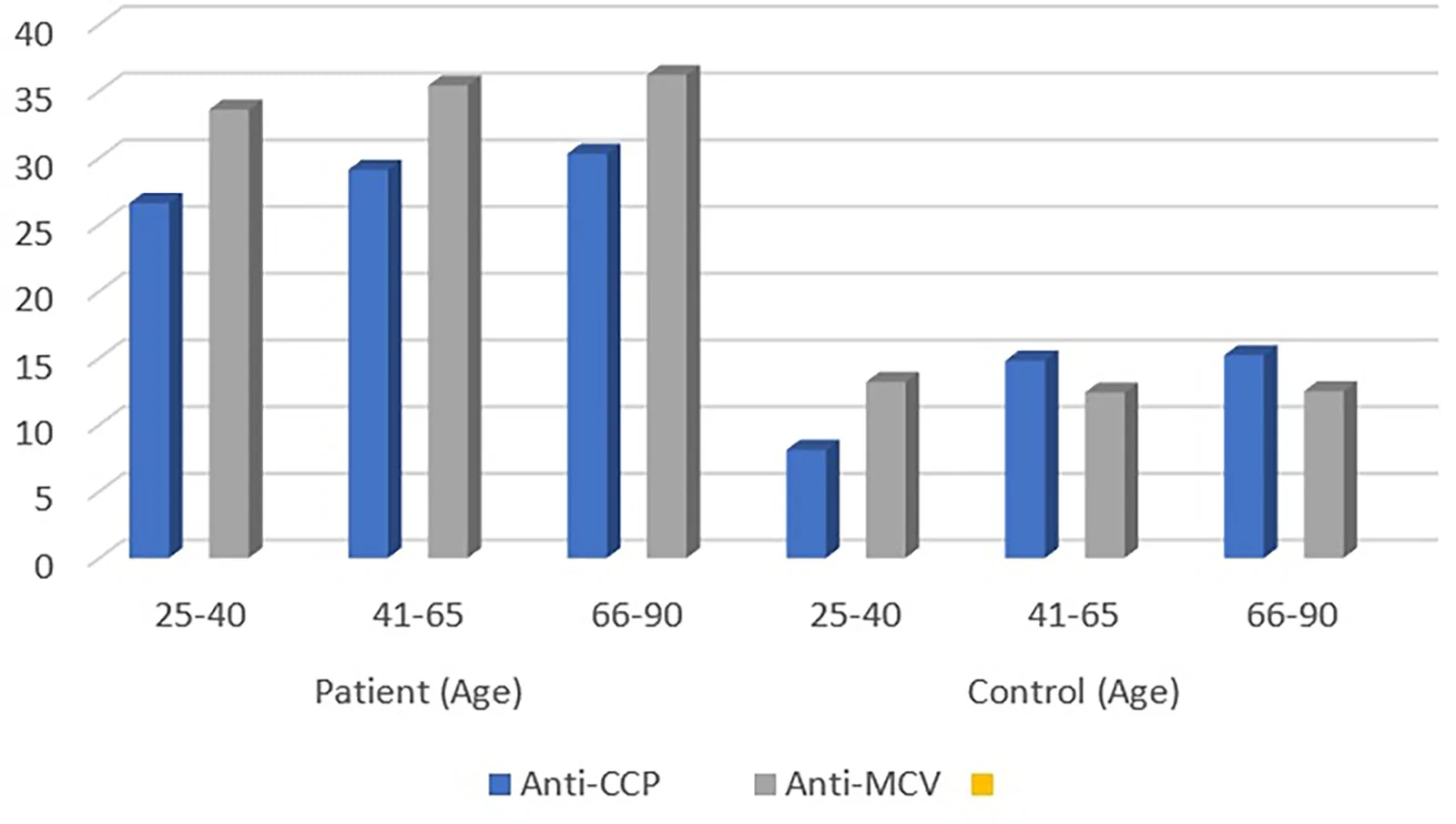
Level of anti-CCP and Anti-MCV in RA patients compared with control.

Findings for anti-CCP and anti-MCV across groups of RA patients (25-40 years, 41-65 years, and 66-90 years), and corresponding control groups are reported in Table 3. Compared to their control subgroups, anti-CCP and anti-MCV levels in all subgroups of RA patients were significantly higher (P < 0.0001). Nevertheless, there was no statistically significant variation observed among the subgroups of RA and each of the control groups for the specified biomarkers, as shown in Figure 3.

**Table 3.** The concentration of anti-cyclic citrullinated peptide (anti-CCP) and anti-mutated citrullinated vimentin (anti-MCV) of patients with rheumatoid arthritis and controls for different subgroups of age.

Parameter	Groups						P value
	Patient (Age)			Control (Age)			
	25-40	41-65	66-90	25-40	41-65	66-90	
	Means±SD	Means±SD	Mans±SD	Means±SD	Means±SD	Means±SD	
** Anti-CCP (U/ml)**	26.6±6.68	29.1±6.5	30.3±5.4	8.1±2.3	14.8±3.0	15.2±3.1	<0.0001 *
**Anti-MCV (U/ml)**	33.6±5.4	35.4±5.7	36.2±5.9	13.2±1.9	12.4±5.2	12.5±2.7	<0.0001 *

Anti-CCP = anti-cyclic citrullinated peptide, anti-MCV = anti-mutated citrullinated vimentin.

* Indicate statistically significant differences at P < 0.0001.

**Figure 4.**
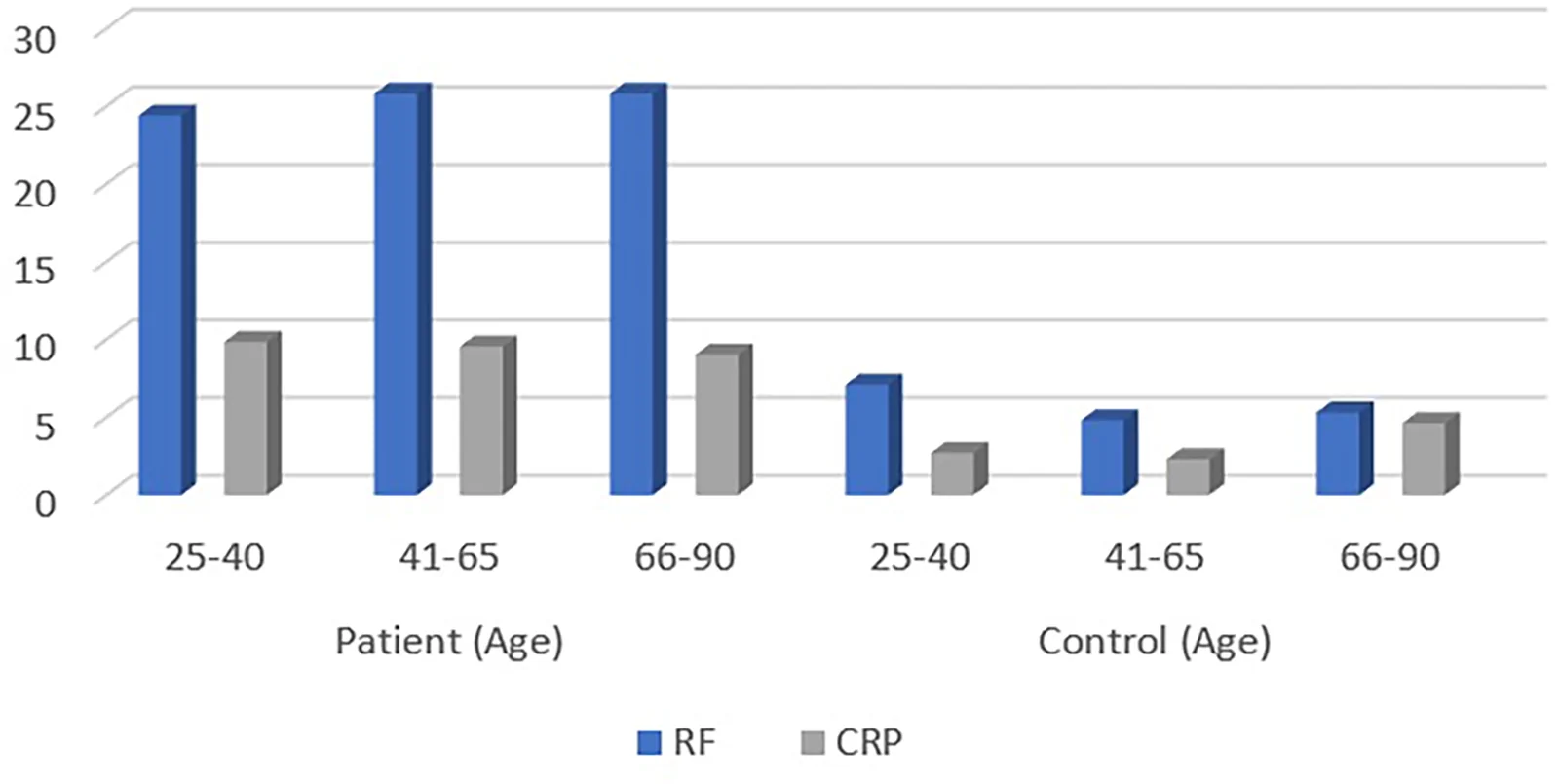
Levels of RF and CRP in RA patients compared with control.

Table 4 and Figure 4 presents data on the levels of RF and CRP across subgroups of RA patients (25-40 years, 41-65 years, and 66-90 years), and their corresponding control groups. The results suggest that for all RA subgroups, both RF and CRP levels showed significant elevation (P < 0.0001) compared to control subgroups. Whereas, the RA and control groups showed that the difference between subgroups did not reach statistical significance for these inflammatory markers.

**Table 4.** The concentration of Rheumatoid Factor (RF) and C-Reactive Protein (CRP) of patients with rheumatoid arthritis (RA) and controls for different subgroups of age.

Parameter	Groups						P value
	Patient (Age)			Control (Age)			
	25-40	41-65	66-90	25-40	41-65	66-90	
	Means±SD	Means±SD	Mans±SD	Means±SD	Means±SD	Means±SD	
** RF (IU/ml)**	24.4±3.91	25.8±4.98	25.8±6.30	7.1±0.96	4.8±2.32	5.3±2.46	<0.0001 *
**CRP (mg/L)**	9.84±1.60	9.55±1.71	9.02±1.91	2.71±1.34	2.29±0.94	4.61±1.74	<0.0001 *

RF = Rheumatoid Factor, CRP = C-Reactive Protein.

* Indicate statistically significant differences at P < 0.0001.

## Discussion

This study assessed critical biomarkers such as ESR, IgG, IgM, anti-CCP, anti-MCV, RF, and CRP in patients with RA who were 25-40, 41-65, 66-90 years and compared with age-matched controls. Research findings showed elevations in all biomarkers which mean patients with RA at all ages have systemic inflammation and autoimmune dysregulation. These findings contribute to our understanding of the clinical utility in the diagnosis and monitoring of RA pathophysiology. This work analyzes some of the diagnostic inflammation, immunology, and hematology marker panels aimed to refine the diagnosis and therapy of RA, and is anchored on the recent biomarker breakthroughs reshaping the management of RA ( Sahin et al., 2025). Among other important markers, C-reactive protein and the ESR, the gold standards in the clinical assessment of inflammation and the disease activity of RA ( Masoumi et al., 2024), remains important in the management of RA.

In RA, the ESR is raised because of the presence of acute-phase proteins (e.g. fibrinogen and immunoglobulins), that enhance the aggregation of red blood cells (RBCs) in which the ESR is a surrogate marker. This elevation not only indicates systemic inflammation, but also reflects inflammatory complications such as heart failure in RA ( Maradit-Kremers et al., 2007) which emphasizes the important role the ESR has in assessing the inflammatory burden.

An ESR level of ≥100 mm/h indicates a high probability of associating with active rheumatic conditions such as RA and vasculitis, specifically in conjunction with active disease exacerbations. Although ESR lacks specificity with RA, its value in monitoring disease state and adjusting treatment is clear in the persistent elevation the disease state. In the RA population, high values continue to validate its role and importance as a measure of inflammation with a direct relationship to disease activity ( Özsoy et al., 2022; Lee et al., 2017).

Elevated levels of IgG and IgM in RA patients provide evidence that the B-cell compartment is hyperactive, further reinforcing the contributions of humoral immunity to the RA pathogenesis. Autoimmunity is mediated by immunoglobulins, and the increase of these molecules marks the activation of the immune system ( Choe et al., 2023). The lack of important age differences indicates that the markers may be even used as stable biomarkers in RA as their values remain high in all age groups.

Increased IgG and IgM in RA patients indicates dysregulation of the B-cell compartment and autoantibody production that contributes to the pathogenesis of RA. The presence of IgM, and in particular rheumatoid factor, is a classical serological marker for RA with 70–80% of patients positive for IgM-RF ( Nicolò et al., 2022).

IgM-RF contributes to the formation of immune complexes which leads to inflammation of the synovium and destruction of the joint, worsening the severity of the condition. More importantly, IgM levels are much higher in RA patients with vasculitis compared to those with RA in its uncomplicated form, indicating its importance in the more serious disease phenotypes and associated comorbidities ( Aiman et al., 2020).

The joint elevation of ESR with IgG and IgM illustrates the connection between systemic inflammation and the autoimmune destruction of tissues in RA. These markers are of great importance for diagnosis and offer great prognostic value. Similarly, as seen in the case of rheumatoid arthritis, IgM, and specifically IgM rheumatoid factor, is one of the most prominent serological markers for RA ( Nicolò et al., 2022).

Anti-CCP antibodies are the most specific and sensitive markers for the diagnosis of RA and help in distinguishing the disease from other arthritic conditions ( Aridoğan et al., 2008). Patients with anti-CCP antibodies and RA show more severe bone erosion and greater radiographic progression relative to patients with RA disease who are anti-CCP negative.

When used in conjunction with anti-CCP, anti-cyclic citrullinated SR-A peptide (anti-CSP) has shown to provide better diagnostic accuracy, particularly in early, seronegative cases of RA ( Xie et al., 2025). The findings of this study in conjunction with the findings of Vittecoq et al. (2003) in which their findings were suggestive of the RF and anti-CCP antibodies concentration being elevated and of significant correlation with more severe manifestations of the disease in early rheumatoid arthritis the disease, which is consistent with the report in question, assists to confirm and support the findings of this study.

Anti-MCV antibodies have comparable diagnostic ability with anti-CCP and RF and so are clinically relevant in the diagnosis of RA. Some studies have shown that anti-MCV could be used as a predictor of disease progress by correlating it with disease activity and predicting joint deformity. Moreover, the disease progress prediction, along with its disease activity and RA in advanced stages prediction, makes anti-MCV a more reliable RA marker than others, especially in tracking progress and monitoring response to treatment ( Zhu et al., 2019). Anti-MCV also has comparable diagnostic performance as anti-CCP in RA, and correlates better with disease activity.

Recent studies show that anti-MCV might be a more sensitive indicator of anti-CCP antibodies of the radiologic joint damage ( Jilani and Mackworth-Young, 2015).

Rheumatology departments now regard the anti-CCP and the anti-MCV as one of the most important and specific markers of RA. These autoantibodies are clinically tied to more aggressive forms of the disease, including rapidly progressive arthritis and severe extra-articular disease ( Nell et al., 2005). We continue to affirm the diagnostic potential of these indicators, esp. anti-CCP, which still holds a major tell-tale specificity for identifying RA, even in its early stages ( Nielen et al., 2004).

RF remains one of the most important and foundational diagnostic criteria for RA. It is present in 70-80% of the population who have RA and still holds its place as a major indicator, even if weak and non-specific. Elevated titers of IgM-class Rheumatoid Factor are associated with negative prognostics. In the case of RA, the autoimmunogenic consequences of RF are exacerbated by the autoimmune complex triggering toxic synovitis and arthritis ( Martinez-Prat et al., 2018). As a component of systemic inflammation in RA, CRP is also of primary importance as a non-specific measure of the disease.

Research is clear that elevated CRP inflammatory levels are associated with increased disease activity and likelihood of comorbidities, particularly cardiovascular disease. Due to CRP’s unique capability of tracking inflammation levels, it has become a crucial component in the management of RA ( Pope & Choy, 2021). Patients with chronic RA also have elevated CRP levels demonstrating its value as an inflammatory biomarker. In rheumatoid arthritis, CRP levels strongly associated with disease activity and deteriorating joints further reiterates the importance of CRP in management and response to treatment ( Aletaha & Smolen, 2018). In rheumatology practice, the incorporation of routine comprehensive biomarker profiling and management of RA with ESR, CRP, RF, anti-CCP, and anti-MCV has the potential to considerably change the discipline. The ability to identify abnormal markers and begin treatment could avert irreversible joint damage and improve the long-term clinical course.

In addition, ongoing assessment of these indicators helps ascertain objectively whether there is treatment progress being made with conventional and biologic disease-modifying antirheumatic drugs (DMARDs) and with the use of these DMARDs, enabling the adoption of precision medicine in the treatment of RA ( Colina & Campana, 2025). This study has some limitations that should be noted, including its limitation to male participants only, which restricts the generalizability of the results to the entire population. Furthermore, the samples were collected from one city, which may affect the overall representativeness of the population. Despite these limitations, this study provides valuable and important information about the hematological and immunological changes associated with RA.

## Conclusion

The range of influences on age regarding alterations to autoimmune and blood parameters in different forms of arthritis can be divergent. The elevations of ESR and systemic inflammation and autoimmune markers such as IgG, IgM, IgA, anti-CCP, anti-MCV, RF, and CRP ensure that there is no escape from chronic inflammation and autoimmune dysregulation that is indicative of RA. The described alterations also signify the need for further exploration of these parameters in the context of diagnosis and for tailoring future treatment to the needs of an individual. Maximizing the use of these elements in routine care should allow for the ideal management of RA to limit the risk of irreversible damage to the joints. Further investigations aimed at clinical integration of these markers would support improvements in patient management, and the enduring consequences of RA would be lightened for many individuals.

## Ethics approval

All procedures involving human participants were performed in accordance with the ethical standards of the institutional research committee and with the 1964 Declaration of Helsinki and its later amendments. The study has been approved by the Ethics Committee of (UOF.MED.05-240911) Medicine - Fallujah University, Iraq.

All participants were adults and provided written informed consent before enrollment. The Ethics Committee approved the written consent procedure.

## Consent to publish

All authors have reviewed and approved the final version of the manuscript and consent to its publication.

## Data Availability

Due to ethical restrictions related to participant confidentiality and as mandated by the Ethics Committee of Fallujah University (UOF.MED.05-240911), the raw participant-level data cannot be openly shared. The IRB approved the study on the condition that data would be accessible only to qualified researchers upon reasonable request for ethically approved scientific purposes. Access requests may be directed to the corresponding author and will require approval from the Ethics Committee. Data will be shared in de-identified form under a data-use agreement.
