# Supplementary material for: Physiological, Immunological, and Erythrocyte Sedimentation Rate Alterations in Male Patients with Rheumatoid Arthritis
Source: F1000Res. 2026 Aug 27;14:1458. Originally published 2025 Dec 26. [Version 2] doi: 10.12688/f1000research.174118.2 (PMC13560013; doi:10.12688/f1000research.174118.2)
Supplement: Peer review history

# Reviewer report: Reviewer response for version 2

**DOI:** 10.5256/f1000research.209098.r515180

**Published:** 2026-08-28

**Authors:** Thikra Muhammed

**Affiliations:** 1 University of Anbar, Ramadi, Iraq

I have reviewed all the comments and concur with them; I recommend  indexing  the research.

Is the work clearly and accurately presented and does it cite the current literature?

Yes

If applicable, is the statistical analysis and its interpretation appropriate?

Yes

Are all the source data underlying the results available to ensure full reproducibility?

Yes

Is the study design appropriate and is the work technically sound?

Yes

Are the conclusions drawn adequately supported by the results?

Yes

Are sufficient details of methods and analysis provided to allow replication by others?

Yes

Reviewer Expertise:

My area of ​​specialization is immunology, and I also have an interest in physiology

I confirm that I have read this submission and believe that I have an appropriate level of expertise to confirm that it is of an acceptable scientific standard.

# Reviewer report: Reviewer response for version 1

**DOI:** 10.5256/f1000research.191995.r510792

**Published:** 2026-08-19

**Authors:** Thikra Muhammed

**Affiliations:** 1 University of Anbar, Ramadi, Iraq

**The research is good, but it requires some adjustments to become a complete piece of work.**

**Weaknesses and Points Needing Revision**

 1-Title changed to(Immunological and ESR Alterations as Biomarkers in  males with Rheumatoid Arthritis"

 2- in Materials and methods This phrase is to be deleted. (with the aim of obtaining more accurate and clear results, with future studies to include a comparison between the sexes)

 and is replaced by (Collected from males only

 3- in  This phrase

 (Participants with other chronic diseases, such as cardiovascular, hypertension, diabetes type I, II, or any other chronic autoimmune diseases, were excluded from this study. Blood samples (5 ml) were collected in a gel tube and left at room temperature for 15 min. Blood tubes were centrifuged at 860g for 10 min. Serum was collected and stored at -20 °C until used. Parameters were measured by fully automated immunological tests by Alegria® ORGENTEC – Germany for (anti-CCP Ab, anti-MCV Ab, Rheumatoid Factor titration). The total immunoglobulin screening (IgG & IgM) and C-Reactive Protein tests were measured by Dimension® EXL 200 – Germany. ESR was measured by manual protocol. The experiment was conducted during the period from July 2023 to October 2023(.

**It is preferable to state the names of the techniques used to measure the variables, rather than simply listing the names of the devises .**

**4- **In Results  ( significantly older) It replaces  by s ignificantly increase wherever it is mentioned **.**

 5- The SD value is very high !

 6-Since the groups have been divided into age categories, it is preferable to compare the variables across these different categories to examine the effect of age.        

Is the work clearly and accurately presented and does it cite the current literature?

Yes

If applicable, is the statistical analysis and its interpretation appropriate?

Yes

Are all the source data underlying the results available to ensure full reproducibility?

Yes

Is the study design appropriate and is the work technically sound?

Yes

Are the conclusions drawn adequately supported by the results?

Yes

Are sufficient details of methods and analysis provided to allow replication by others?

Yes

Reviewer Expertise:

My area of ​​specialization is immunology, and I also have an interest in physiology

I confirm that I have read this submission and believe that I have an appropriate level of expertise to confirm that it is of an acceptable scientific standard, however I have significant reservations, as outlined above.

# Response

**Published:** 2026-08-20

**Authors:** Mohammed AlHamadani

**Affiliations:** Department of Biology, College, University of Fallujah, Al-Fallujah, Al Anbar Governorate, Iraq

**Dear Reviewer2 Dr. Thikra Muhammed,** We sincerely thank you for your careful evaluation of our manuscript and for your constructive and valuable comments. We have carefully considered each point and have revised the manuscript accordingly. The revisions were undertaken to improve the methodological clarity, statistical interpretation, and overall scientific presentation of the study. Our detailed point-by-point responses are provided below. All corresponding changes have been incorporated into the revised manuscript and highlighted using track changes.  

**Comment 1** **Reviewer comment:** The title should be revised to emphasize the immunological and ESR alterations in male patients with rheumatoid arthritis.

**Response:** We sincerely thank the reviewer for this helpful suggestion. We agree that the study population should be explicitly identified in the title. Accordingly, the title has been revised to: **“Physiological, Immunological, and Erythrocyte Sedimentation Rate Alterations in Male Patients with Rheumatoid Arthritis.”** This revised title more accurately reflects the scope of the study and clearly identifies the study population. We have also ensured that the restriction of the study population to male participants is clearly stated in the Abstract and Materials and Methods sections.      

**Comment 2** **Reviewer comment:** In the Materials and Methods section, the statement explaining the selection of males should be deleted and replaced by a concise statement indicating that participants were male only.

**Response:** We thank the reviewer for this clarification and fully agree. The previous wording has been revised to provide a more concise and scientifically neutral description of the study population. The Materials and Methods section now states: “In this study, a total of 90 male subjects were recruited from Fallujah Teaching Hospital and various diagnostic laboratories. Only male participants were included to maintain a relatively homogeneous study population and to minimize potential sex-related biological variability in hematological and immunological parameters.” We have also acknowledged the limitation associated with the male-only study population in the Discussion section, particularly with respect to the generalizability of the findings to female patients.  

**Comment 3** **Reviewer comment:** In Materials and Methods, it is preferable to state the names of the techniques used to measure the variables rather than simply listing the names of the devices.

**Response:** We sincerely appreciate this valuable methodological suggestion. We agree that describing the analytical methodology in addition to the instrument names improves reproducibility and methodological transparency. Accordingly, the Materials and Methods section has been revised to specify the analytical approaches used for each parameter: “Parameters including anti-CCP Ab, anti-MCV Ab, and rheumatoid factor (RF) titration were quantitatively determined using automated immunoassay-based methods on the Alegria® system (ORGENTEC Diagnostics GmbH, Germany). Total immunoglobulin G (IgG), immunoglobulin M (IgM), and C-reactive protein (CRP) were measured using particle-enhanced turbidimetric immunoassay methods on the Dimension® EXL 200 Integrated Chemistry System (Siemens Healthineers, Germany). Erythrocyte sedimentation rate (ESR) was determined manually using the standard Westergren method.” This revision provides greater methodological detail while retaining the information regarding the analytical systems used.  

**Comment 4** **Reviewer comment:** In the Results section, “significantly older” should be replaced by “significantly increased” wherever it is mentioned.

**Response:** We thank the reviewer for identifying this wording issue. We have carefully reviewed the Results section and revised the terminology where appropriate. Importantly, the wording has been adjusted according to the parameter being described. Expressions referring specifically to age are reported as differences in age, whereas expressions referring to biomarker concentrations or other measured parameters use “higher” or “increased,” as appropriate. This revision improves the statistical and scientific precision of the Results section.  

**Comment 5** **Reviewer comment:** The SD value is very high.

**Response:** We appreciate the reviewer’s important observation. The relatively large standard deviations observed for some immunological and inflammatory biomarkers, particularly RF, anti-CCP, and CRP, indicate substantial inter-individual variability within the study population. Such variability may reflect differences in the magnitude of immune activation and inflammatory responses among patients with rheumatoid arthritis, even though all patients were newly diagnosed and treatment-naïve. We have therefore avoided interpreting the standard deviation solely as a statistical anomaly and have instead discussed the observed variability in the context of biological heterogeneity among patients. Where appropriate, the revised statistical analysis now includes assessment of the underlying data distribution, and the Statistical Analysis section has been revised to accurately describe the statistical procedures actually applied. We have also added an explanatory statement to the Discussion to clarify the possible biological basis of the observed variability.  

**Comment 6** Since the groups have been divided into age categories, it is preferable to compare the variables across these different categories to examine the effect of age. ****Response:** We thank the reviewer for highlighting this point. The age distribution and baseline comparison across different age subgroups between Rheumatoid Arthritis (RA) patients and controls are already evaluated and presented in Table 1 and its accompanying text in the Results section.** ****Revision in Manuscript:** To further clarify the purpose of this subgroup division and address the reviewer's comment, we have added the following sentence to the text preceding Table 1 in the Results section:** **"Furthermore, this structured subgroup stratification provides a baseline framework to ensure clear age categorization and to evaluate baseline parameter variations across specific age categories."** **Additional clarification regarding study limitations** In response to the reviewer’s comments and to improve transparency, we have further strengthened the Discussion by explicitly acknowledging the principal limitations of the study, including the male-only population, recruitment from a single geographic region, the exploratory nature of the investigation and absence of an a priori power calculation, and the cross-sectional design. These limitations are now clearly stated so that the findings are interpreted within the appropriate clinical and methodological context.  

**Closing response** We are grateful to the reviewer for these constructive comments. We believe that the revisions have substantially improved the methodological transparency, statistical interpretation, and scientific clarity of the manuscript. We sincerely appreciate the time and expertise devoted to reviewing our work. Kind regards, **Assoc. Prof. Dr. Mohammed Y.I. Al-Hamadani** Corresponding Author Department of Biology, College of Education, University of Fallujah, Iraq

# Reviewer report: Reviewer response for version 1

**DOI:** 10.5256/f1000research.191995.r475310

**Published:** 2026-05-20

**Authors:** Ali Mohammed Abed

**Affiliations:** 1 biology, University of Tikrit, Tikrit, marge, Iraq

Summary of the Article

 This study investigates hematological and immunological biomarkers in male rheumatoid arthritis (RA) patients compared to healthy controls. The authors analyzed erythrocyte sedimentation rate (ESR), immunoglobulins (IgG, IgM), anti-cyclic citrullinated peptide (anti-CCP), anti-mutated citrullinated vimentin (anti-MCV), rheumatoid factor (RF), and C-reactive protein (CRP) across three age groups (25–40, 41–65, 66–90 years).

**Key findings:**

 Significant elevations in ESR, IgG, IgM, anti-CCP, anti-MCV, RF, and CRP in RA patients compared to controls (p < 0.0001).

 Biomarker elevations were consistent across age groups, suggesting systemic inflammation and autoimmune dysregulation independent of age.

 Authors conclude that these markers can improve diagnostic accuracy, guide personalized treatment, and prevent joint damage.

 Strengths

 Clear clinical relevance: The study highlights biomarkers widely used in RA diagnosis and monitoring.

 Structured methodology: Patient selection followed ACR/EULAR 2010 criteria, ensuring diagnostic accuracy.

 Comprehensive biomarker panel: Inclusion of ESR, CRP, RF, anti-CCP, and anti-MCV provides a robust diagnostic framework.

 Statistical rigor: Use of GraphPad Prism and clear reporting of p-values strengthens validity.

 Discussion integrates literature: The authors contextualize findings with prior studies, reinforcing credibility.

**Weaknesses and Points Needing Revision**

 1. Study Population Limitations

 Only male participants were included. While justified (to avoid hormonal confounders), this severely limits generalizability.

 Constructive suggestion: Future studies should include female participants and analyze sex-based differences, as RA disproportionately affects women.

 2. Sample Size and Representativeness

 The study recruited 90 participants from a single city (Fallujah). This restricts external validity.

 Suggestion: Expand recruitment to multiple centers and increase sample size to strengthen representativeness.

 3. ESR Reporting Issues

 Table 2 shows ESR values for patients and controls that appear very close (e.g., 33.4 vs. 30.9 mm/hr in young adults), yet reported as highly significant (p < 0.0001).

 Suggestion: Clarify whether these values reflect true differences or if statistical reporting needs correction. Consider including effect sizes, not just p-values.

 4. Age Differences

 The results section notes “significant age-related differences” in RA and control groups, but no clear biological interpretation is provided.

 Suggestion: Expand discussion on whether age influences biomarker variability or disease severity.

 5. Discussion Depth

 While the discussion references literature, it sometimes repeats known facts (e.g., CRP as a non-specific inflammation marker) without linking directly to study findings.

 Suggestion: Strengthen the discussion by explicitly connecting this study’s results to clinical implications (e.g., how elevated IgM/IgG could guide treatment decisions).

 6. Figures and Tables

 Some tables/figures contain formatting inconsistencies (e.g., “Means+SD” vs. “Means±SD”).

 Suggestion: Standardize formatting and ensure clarity in figure legends.

 7. Ethical and Data Transparency

 The ethics approval is clearly stated, but raw data is unavailable due to confidentiality.

 Suggestion: Provide anonymized aggregated datasets or supplementary material to allow independent verification.

 Points That Must Be Addressed for Scientific Soundness

 Clarify ESR results – ensure statistical reporting matches observed differences.

 Address gender limitation – acknowledge explicitly in discussion and propose inclusion of females in future work.

 Expand on age-related findings – provide biological interpretation of differences.

 Improve discussion linkage – connect biomarker elevations directly to clinical management strategies.

 Standardize tables/figures – correct formatting inconsistencies.

 Enhance data transparency – provide supplementary aggregated data if possible.

 Overall Recommendation:

 The article presents valuable findings on RA biomarkers and contributes to clinical understanding. However, to be scientifically sound and suitable for indexing, the authors must clarify statistical reporting, address population limitations, and strengthen discussion of clinical implications.

 With these revisions, the study could serve as a strong reference for biomarker-based RA diagnosis and monitoring.

Is the work clearly and accurately presented and does it cite the current literature?

Yes

If applicable, is the statistical analysis and its interpretation appropriate?

Yes

Are all the source data underlying the results available to ensure full reproducibility?

Partly

Is the study design appropriate and is the work technically sound?

Yes

Are the conclusions drawn adequately supported by the results?

Partly

Are sufficient details of methods and analysis provided to allow replication by others?

Yes

Reviewer Expertise:

immunological

I confirm that I have read this submission and believe that I have an appropriate level of expertise to confirm that it is of an acceptable scientific standard.
